# Maternal mental health mediates links between socioeconomic status and child development

**DOI:** 10.1007/s12144-022-03181-0

**Published:** 2022-06-09

**Authors:** Tess A. Smith, Rogier A. Kievit, Duncan E. Astle

**Affiliations:** 1 MRC Cognition and Brain Sciences Unit, University of Cambridge, 15 Chaucer Road, Cambridge, UK; 2 Donders Institute for Brain, Cognition and Behaviour, Radboud University, Nijmegen, Netherlands

**Keywords:** ALSPAC, Socioeconomic status, Maternal mental health, Child mental health, Child cognitive ability

## Abstract

The impact of socioeconomic status (SES) on early child development is well-established, but the mediating role of parental mental health is poorly understood. Data were obtained from The Avon Longitudinal Study of Parents and Children (ALSPAC; *n* = 13,855), including measures of early SES (age 8 months), key aspects of development during mid-late childhood (ages 7–8 years), and maternal mental health during early childhood (ages 0–3 years). In the first year of life, better maternal mental health was shown to weaken the negative association between SES and child mental health. Better maternal mental health was additionally shown to weaken the association between SES and child cognitive ability. These findings highlight the variability and complexity of the mediating role of parental mental health on child development. They further emphasise the importance of proximal factors in the first year of life, such as parental mental health, in mediating key developmental outcomes.

## Introduction

As of 2020, 4.3 million children living in the UK are growing up in a low-income household (British Government Department of Work and Pensions, 2021). The impact of poverty on development is well-established, with childhood deprivation associated with negative outcomes for brain architecture, physiological and psychological health, cognitive development, educational attainment, and socioemotional well-being (Evans & Kim, 2013; Hair et al., 2015; Luby et al., 2013; Reiss et al., 2019). These associations start as early as infancy and continue into adulthood (Blair & Raver, 2016; Chen et al., 2002; Tooley et al., 2021). Parental income does not influence development in isolation, but in combination with factors such as parental educational and occupational status, as part of a broader construct called socioeconomic status (SES).

### Challenges Faced by the Literature

The association between early SES and a child’s development remains complex, and there are notable challenges faced by the literature to date. Addressing these challenges provides an opportunity to expand our understanding of SES-outcome associations. The first opportunity comes with including multiple different outcome measures. There have been a large number of studies that have taken single outcome domains and explored pairwise associations with socioeconomic variables (Bøe et al., 2014; Fitzsimons et al., 2017; Kinge et al., 2021; Melchior et al., 2012; Reiss, 2013; Reiss et al., 2019; Wickham et al., 2017). Considering single developmental domains alone makes it difficult to determine whether SES-outcome associations are domain specific or domain general. For example, the scope for explaining SES-childhood health outcomes depends on the health behaviour in question, with stronger associations between injury and low SES in early childhood, and smoking behaviour and low SES in adolescence (Chen et al., 2002). Systematic reviews and meta-analyses further demonstrate that the strength of the association varies across developmental domains, with stronger associations seen within language and cognitive domains, compared to physical and psychological health, emotional maturity, communication skills, and general knowledge (Letourneau et al., 2013; Webb et al., 2017). These findings suggest that it could be important to consider multiple developmental domains simultaneously, in order to establish the specificity of any SES associations.

Correspondingly, a second opportunity to further explore the association between SES and developmental outcomes, comes with the consideration of mediating factors. An additional challenge present in the current literature is the scarcity of studies investigating plausible mediating factors. This is problematic, as the complexity of SES-outcome associations is likely in part because they are not direct, but rather the result of multiple intricate processes, such as mediating paths. In this way, the overall effect of SES may depend on specific mediating factors in a child’s proximal environment, which have downstream consequences for development (Chen, 2004; Chen & Miller, 2013; Letourneau et al., 2013; Liu et al., 2020; Luby et al., 2013). Likewise, pathways operating at the level of a child’s neighbourhood, family, individual self, and biology, have all been linked to low SES and child health outcomes (Chen & Miller, 2013). Thus, incorporating potential mediators is crucial if we want to unpack the mechanisms that help explain SES-outcome associations.

### Potential Mediating Role of Maternal Mental Health

One plausible candidate mediating associations with SES is parental mental health. Persistent and transitions into poverty have been associated with an increased risk of maternal mental health difficulties (Fitzsimons et al., 2017; Wickham et al., 2017). In turn, children whose mothers experience mental ill-health during their primary education are at a higher risk of developing mental health problems themselves during this time (Fitzsimons et al., 2017). Correspondingly, maternal depression increases the risk of cognitive difficulties in early infancy (Liu et al., 2017). Given the emerging literature demonstrating a strong association between early SES and a child’s mental health and cognitive development, as well as associations between these measures and maternal mental health, it seems plausible that maternal mental health may mediate the association between SES and child outcomes. Whether maternal mental health amplifies or buffers the effect of poverty on development is a question with little empirical evidence. Of the few studies that have considered the mediating role of maternal mental health, few consider multiple developmental domains or longitudinal measures of maternal mental health (Bøe et al., 2014; Melchior et al., 2012; Reiss, 2013; Reiss et al., 2019; Webb et al., 2017).

Combining the two challenges outlined above, it is possible that maternal mental health mediates SES-outcome relationships differentially. For example, according to the dimensional model of early adversities (McLaughlin et al., 2014, 2021; Sheridan & McLaughlin, 2014), a prominent theory within this area, different types of adversity are causally associated with different types of outcomes. Socioeconomic disadvantage results in the relative absence of cognitive and social stimulation and is most strongly associated with cognitive developmental outcomes. In contrast, threatening experiences, such as abuse, are most strongly linked with socioemotional outcomes and mental well-being (Sheridan et al., 2020). According to this theory, whilst we might observe significant relationships between maternal and child mental health (Fitzsimons et al., 2017), the specific SES-mediating role of maternal mental health may be stronger for children’s cognitive outcomes. This is simply because SES, an example of ‘deprivation’ within this framework, ought to most strongly be associated with children’s cognitive outcomes (Johnson et al., 2021), and thus there is more to mediate. In short, it is possible that maternal mental health might mediate SES-outcome relationships differentially depending upon the outcome.

A third challenge within the literature is that we do not know whether there are particular windows wherein maternal mental health is *more likely* to have a longitudinal effect on child development. Considering developmental timing is valuable, as there are sensitive periods during development when considerable changes take place. For example, the first three years of life are crucial for brain development, and exposure to adversity during this time may have a disproportionate impact, relative to other developmental periods (Nelson III & Gabard-Durnam, 2020; Ouyang et al., 2019). In addition, substantial and important changes take place during primary education, between the ages of 5 and 11, where cognitive abilities develop rapidly (Spiegel et al., 2021; Van der Ven et al., 2012). This is also the most common time for the onset of mental, behavioural, and developmental difficulties (Robinson et al., 2017). Thus, considering longitudinal measures of maternal mental health in the early years, when the brain is undergoing rapid macro- and microstructural changes, alongside childhood cognitive and mental health during a time of meaningful development, such as during primary education, could provide an opportunity to identify sensitive windows where maternal mental health is most likely to predict risk of childhood mental health and cognitive difficulties. By extension, this could allow for the identification of optimal periods where interventions are likely to have the greatest impact on developmental outcomes.

### The Current Study

The purpose of this study is to attempt to address these three interrelated challenges by incorporating different outcomes, considering maternal mental health as a potential mediator, and testing for mediating effects across multiple developmental periods. We specifically sought to address the following questions: 1) Does maternal mental health act as a longitudinal mediator for the association between SES and common childhood development outcomes, such as child mental health and cognitive ability? 2) If maternal mental health does in fact mediate the association, are there sensitive periods where the mediating effect of maternal mental health, if any, is particularly strong? We used data from The Avon Longitudinal Study of Parents and Children (ALSPAC; Boyd et al., 2013; Fraser et al., 2013), including measures of early SES, key aspects of child development (i.e., child mental health and cognitive ability), and maternal mental health across three waves. In doing so, we aimed to specify the timing of the mediating effect of maternal mental health on these associations by analysing maternal mental health at three different timepoints, as opposed to moderated mediation. Analyses were carried out within a structural equation modelling (SEM) framework (Gana & Broc, 2019; Iacobucci et al., 2007; Schreiber et al., 2006).

## Methods

### Participant Demographic

This study was conducted using data from The Avon Longitudinal Study of Parents and Children (ALSPAC), a transgenerational cohort study based in the region of Avon, England, where 13,761 eligible pregnant women with an expected delivery date between 1^st^ April 1991 and 31^st^ December 1992 were recruited, with a mean age of 28 ranging from 14 to 46 (*SD* = 5.0) (Boyd et al., 2013; Fraser et al., 2013). Of the mothers who participated in ALSPAC, 79.1% lived in owner-occupier accommodation, 90.8% had a car, 79.4% were married, and 2.2% were non-White. By the third phase of recruitment, data were collected from 14,009 children by way of self-report or on behalf of the biological mother or primary caregiver. Of this cohort, 49.2% were female, 86.5% were White, and 12.5% were from a low-income household. The current study comprised 13,855 of this cohort. The data were obtained from children and their biological mothers when the children were aged 8 months, 1 year and 9 months, 2 years and 9 months, 7 years and 5 months, and 8 years.

Ethical approval for the study was obtained from the ALSPAC Ethics and Law Committee and the Local Research Ethics Committees. Informed consent for the use of data collected via questionnaires and clinics was obtained from participants following the recommendations of the ALSPAC Ethics and Law Committee at the time. For information pertaining to the ALSPAC Research Ethics Committee policies and supporting documentation, please see https://www.bristol.ac.uk/alspac/researchers/research-ethics/. Please note that the study website contains details of all the data that is available through a fully searchable data dictionary and variable search tool (http://www.bristol.ac.uk/alspac/researchers/our-data/).

### Measures

#### Socioeconomic Status

Socioeconomic status (SES) was assessed by asking mothers to report their social class based on their occupation by selecting one of the following response options: (1) professional; (2) managerial and technical; (3) skilled non-manual; (4) skilled manual; (5) partly skilled; (6) unskilled. Mothers and their partners were further asked to report their level of education by selecting one of the following response options: (0) none; (1) CSE; (2) vocational; (3) O level; (4) A level; (5) degree. Mothers were additionally asked to report the level of difficulty they experienced affording food and paying their rent or mortgage by selecting one of four responses for each scale, ranging from (1) very difficult to (4) not difficult. Reports of social class based on occupation were reverse coded so that higher values represented greater SES, thereby keeping the directionality of responses consistent across all measures of SES. All SES measures were taken at the first timepoint.

#### Maternal Mental Health

Maternal mental health was measured across three waves, when the index children were aged 8 months, 1 year and 9 months, and 2 years and 9 months. At each timepoint, maternal anxiety and depression were measured using the Anxiety and Depression subscales of the Crown-Crisp Experiential Index (CCEI; Birtchnell et al., 1988), a valid, reliable (*k* = 0.88), and widely used self-reported measure of psychopathology. Anxiety and depression subscales of the CCEI comprised a summary score from 8-items and included questions such as “Do you get troubled by dizziness or shortness of breath?” and “Do you feel uneasy and restless”. These items were recoded to a 16-point scale, ranging from (0) not anxious or depressed to (16) very anxious or depressed. Depressive symptoms were further measured using the 10-item Edinburgh Postnatal Depression Scale (EPDS; Cox et al., 1987), which has demonstrated a split half reliability of 0.88. Items from the EPDS (e.g., “I have felt scared or panicky for no very good reason” and “I have been so unhappy that I have had difficulty sleeping”) were recoded to a 29-point scale, ranging from (0) not depressed to (29) very depressed. This globally administered measure of depression during the perinatal period has been shown to be sensitive to changes in depression in both men and women over time (Cox, 2017; Matthey et al., 2001). Maternal mental health scales were recoded so that lower scores were indicative of poorer mental health. Because the respondent for ALSPAC is almost always the mother, data on paternal mental health were not available (Fraser et al., 2013). No maternal mental health data were available prior to the 8 month data collection.

#### Child Mental Health

Child mental health was measured when children were 7 years and 5 months old using symptoms of generalised anxiety, any anxiety disorder, and depression, as reported by parents during a face-to-face clinical assessment and measured using the respective subscales of the Development and Well-Being Assessment (DAWBA; Goodman et al., 2000). Following parent responses to standardised questions during the clinical assessment (e.g., presence and severity of symptoms and the length of time the symptoms have been present) the DAWBA draws upon a computerised algorithm to generate bands based on symptom severity. These bands denote the likelihood of a child having the disorder in question and have been shown to be comparable to clinical-generated diagnoses across varied populations (*k* = 0.63—0.94) (Aebi et al., 2012; Goodman et al., 2011). Child mental health scales were recoded so that lower scores were indicative of poorer mental health. An alternative possibility was to test for relationships between SES, maternal mental health, and children’s behavioural problems. However, we instead opted to use the measures from the DAWBA simply because they are the closest data we have to clinical information on child mental health and are more directly aligned with our measures of maternal mental health.

#### Child Cognitive Ability

**Fig. 1:**
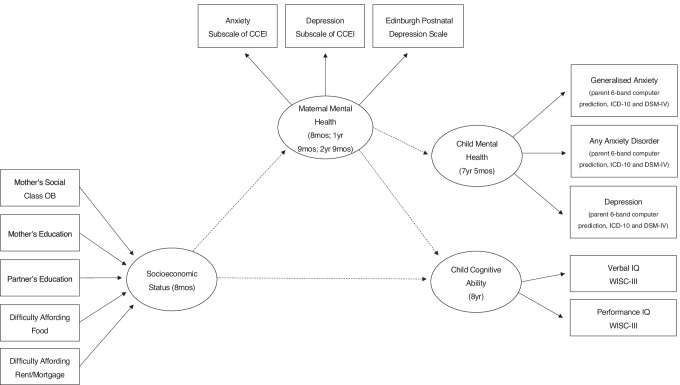
Summary of measures. *Note.* Figure displays all measures included in the present study, including the timepoints at which the data were collected

Cognitive ability was measured at 8 years using Verbal and Performance IQs from the widely used Wechsler Intelligence Scale for Children, 3^rd^ edition, which has demonstrated reliability coefficients ranging from 0.94 to 0.97 (WISC-III; Wechsler, 1991). Both scales yield a standard score (*M* = 100, *SD* = 15) by comparing an individual’s scores to those obtained by a representative sample of similarly aged peers. The Verbal scale comprises five subtests including information (i.e., knowledge and long-term memory), similarities (i.e., abstract reasoning and concept formation), arithmetic (i.e., numerical reasoning and computation), vocabulary (i.e., word knowledge), and comprehension (i.e., practical knowledge and social judgement), whilst the Performance scale subtests include picture completion (i.e., visual perception and attention to detail), picture arrangement (i.e., nonverbal reasoning and sequencing), block design (i.e., spatial visualisation and reasoning), object assembly (i.e., visual perception and organisation), and coding (i.e., visual-motor information processing) (Fig. 1).

### Data Collection

All data were collected by ALSPAC. Biological mothers were administered questionnaires relating to socioeconomic status when children were 8 months, maternal mental health when children were 8 months, 1 year and 9 months, and 2 years and 9 months, and child mental health when children were 7 years and 5 months. Children were directly administered the WISC-III at 8 years (Wechsler, 1991). For further information on all questionnaires administered by ALSPAC, please see https://www.bristol.ac.uk/alspac/researchers/our-data/questionnaires/.

### Statistical Analyses

To identify whether maternal mental health mediates the association between SES and key developmental outcomes, (i.e., child mental health and child cognitive ability), we used structural equation modelling (SEM), a multivariate technique which uses path modelling and latent variables to analyse structural relations among variables (Iacobucci et al., 2007; Schreiber et al., 2006). Analyses were performed using the lavaan package (Rosseel, 2012) in the R (R Core Team, 2021) and RStudio (RStudio Team, 2021) programming environments.

**Fig. 2:**
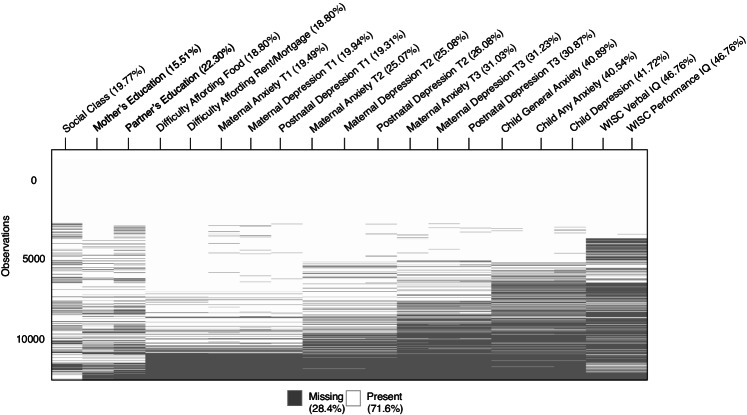
Detailed missingness across study variables. *Note.* Figure displays data missingness prior to imputation for all variables included in the present study

In the first step, we developed an aggregate SES score. As SES is a formative construct, we used principal component analysis (PCA) instead of confirmatory factor analysis (CFA). To allow us to run the PCA with missingness, we used multiple imputation using the Mice package (Buuren & Groothuis-Oudshoorn, 2011). This allowed for a PCA to be carried out to derive one global metric of SES. The resulting global measure of SES was then integrated with the original pre-imputed dataset, and missing data across the full SEM was accommodated using robust full information maximum likelihood (FIML) with Yuan-Bentler scaled test statistic to correct for deviations from multivariate normality (Rosseel, 2012). When estimating missingness in SEM, such as when data are missing at random, FIML has been shown to be superior to alternative methods in that it produces unbiased estimates, higher efficiency, lower convergence failures, and optimal Type-1 error rates (Cham et al., 2017; Enders & Bandalos, 2001). See Fig. 2 for detailed missingness across all variables included in the analysis.

Overall model fit was evaluated using the model chi-square test with its degrees of freedom and *p* value, the Bentler comparative fit index (CFI; Bentler, 1990), the root mean square error of approximation (RMSEA; Steiger, 1990) with its 90% confidence interval, and the standardised root mean square residual (SRMR; Bentler, 1995), respectively. Evaluation of model fit was interpreted as: CFI (acceptable fit ≥ 0.95, good fit ≥ 0.97), RMSEA (acceptable fit ≤ 0.08, good fit ≤ 0.05), and SRMR (acceptable fit ≤ 0.10, good fit ≤ 0.05) (Schermelleh-Engel et al., 2003). To test whether the observed variables (i.e., psychometric assessments of mental health and cognitive ability) could be accurately captured by a set of a prespecified latent variables (i.e., maternal mental health, child mental health, and child cognitive ability, respectively), CFA was carried out (Byrne, 2005). To assess the psychometric equivalence of maternal mental health at each of the three waves, measurement invariance was imposed (Putnick & Bornstein, 2016). Both models were further compared by way of evaluating ΔCFI with a cut-off of < 0.01, to confirm measurement invariance was achieved (Cheung & Rensvold, 2002). Standard errors for the defined parameters were computed in lavaan using the Delta method (Rosseel, 2022). Effect sizes were interpreted using guidelines as recommended by Gignac and Szodorai (2016), with *r* = 0.10, *r* = 0.20, and *r* = 0.30 interpreted as relatively small, typical, and relatively large, respectively.

## Results

### Establishing a Measurement Model

Prior to addressing our specific research questions, a measurement model needed to be established. Achieving this was threefold. Each of these steps and their outcomes will be discussed in turn.

#### Latent Variable Modelling

Firstly, to examine the viability of the latent variables (maternal mental health across three waves, child mental health, and child cognitive ability), that is, specify and test the extent to which the latent variables and the observed variables corresponded to one another; latent variable modelling was carried out with CFA (Byrne, 2005). This allowed us to test whether our hypothesised factor structure was compatible with the data. For identification purposes, the two IQ factor loadings of the WISC indicators were constrained to equality. Goodness of fit indices suggested that the model fit the data well (χ^2^(64) = 778.056, *p* < 0.0001; CFI = 0.991; RMSEA = 0.032 [0.030, 0.034]; SRMR = 0.041; Yuan-Bentler scaling factor = 1.160).

#### Establishing Measurement Invariance

Next, given that maternal mental health was longitudinally measured across three waves, it was essential to establish measurement invariance to ensure the measures were related to the latent construct in the same way at each wave (Widaman et al., 2010). To do so, we equality constrained key indicators (i.e., the same indicators at wave 1-2-3) and compared it to a model where these same parameters were estimated freely. Model comparison showed notably similar results between the freely estimated (χ^2^(64) = 778.056, *p* < 0.0001; CFI = 0.991; RMSEA = 0.032 [0.030, 0.034]; SRMR = 0.041; Yuan-Bentler scaling factor = 1.160) and the constrained model (χ^2^(63) = 502.169, *p* < 0.0001; CFI = 0.994; RMSEA = 0.025 [0.023, 0.027]; SRMR = 0.031; Yuan-Bentler scaling factor = 1.162), with both models fitting the data well. To further establish measurement invariance, the ΔCFI between consecutive models was evaluated and found to be within the designated cut-off point (ΔCFI = 0.003). In other words, imposing the assumption of equal measurement of the latent factors across time did not lead to a substantial reduction in model fit, suggesting measurement invariance was tenable.

#### Developing a Global Metric of Socioeconomic Status

Lastly, whilst the latent variables accounted for thus far (maternal mental health, child mental health, and child cognitive ability) are reflective, in that their association to their allocated observed variables are assumed by CFA to be causal, this is not the case for SES. Unlike the aforementioned variables, this assumption does not hold for SES, which is commonly considered a formative latent variable: An individual’s SES is typically formed by their education, occupation and earnings. To derive a single SES metric, the (rescaled) measures of SES were entered into a PCA. Scores on the first dimension of the PCA, which accounted for 40.01% of the shared variance, were extracted and used as our measure of SES in subsequent analyses. For detailed comparisons among PCA dimension factor loadings, refer to Table 1.

**Table 1:** Factor loadings of SES measures for first PCA dimension

SES measure	DIM1
Parent (1) social class based on occupation	0.45
Parent (1) highest education qualification	0.53
Parent (2) highest education qualification	0.50
Parent (1) difficulty affording food	0.40
Parent (1) difficulty affording rent or mortgage	0.34

*SES* Socioeconomic status; *PCA* Principal component analysis; *DIM* Dimension; Parent (1) = mother; Parent (2) = partner

### Interpretation of the Full Structural Mediation Model

#### Model Fit

**Fig. 3:**
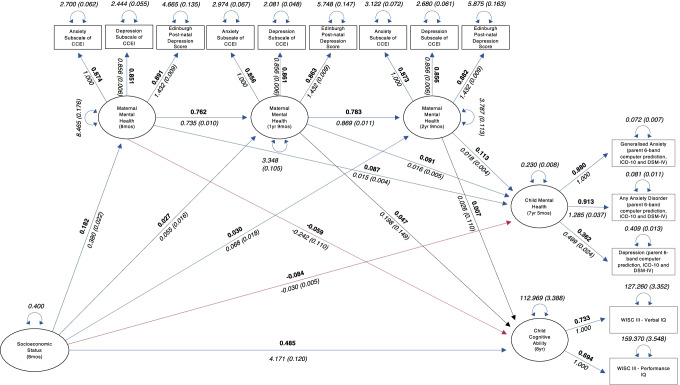
Estimated parameters of the full structural equation model. *Note.* Figure displays analysis output for the full structural equation model. Positive and significant (*p* < 0.05) path estimates are denoted by blue arrows, negative and significant path estimates are denoted by red arrows, and non-significant (*p* > 0.05) path estimates are denoted by dashed arrows. Standardised parameter estimates are in roman, unstandardised parameter estimates (with standard errors in parentheses) are in italics, and key standardised parameters of interest have been displayed in boldface. Curved double arrows display (residual) variances

Once the measurement model was established, the full structural equation model was fit to the data to examine whether maternal mental health mediates the association between SES and key developmental outcomes (i.e., child mental health and child cognitive ability) and to what extent these effects, if any, were present. The full mediation model fit the data well: χ^2^(73) = 1089.607, *p* < 0.0001; CFI = 0.987; RMSEA = 0.034 [0.032, 0.036]; SRMR = 0.037; Yuan-Bentler scaling factor = 1.152. See Fig. 3 for detailed estimated parameters. The mediating effect of maternal mental health on the association between SES and child mental health, and SES and child cognitive ability will be discussed in turn.

#### Mediating Effect of Maternal Mental Health on Child Mental Health

As illustrated in Fig. 3, parameter estimates showed a relatively small but significant negative direct effect of SES on child mental health (*b* = -0.030, *SE* = 0.005, 90% CI = [-0.039, -0.020], β = -0.084, *p* < 0.0001). Children from higher SES households displayed poorer mental health. This association was mediated by maternal mental health. The mediating effect was greatest at 8 months (*b* = 0.006, *SE* = 0.002, 90% CI = [0.003, 0.009], β = 0.016, *p* < 0.0001). Whilst the 1 year 9 months timepoint (*b* = 0.001, *SE* = 0.000, 90% CI = [0.000, 0.002], β = 0.002, *p* < 0.05) and 2 years 9 months timepoint (*b* = 0.001, *SE* = 0.000, 90% CI = [0.000, 0.002], β = 0.003, *p* < 0.005) also showed a significant mediating effect, the mediating effect was primarily accounted for by the 8 month timepoint. The cumulative effect of maternal mental health across all three timepoints resulted in a joint mediation effect: The direct effect of SES on child mental health was considerably attenuated (*b* = -0.022, *SE* = 0.005, 90% CI = [-0.032, -0.012], β = -0.062, *p* < 0.0001). Please see Table 2 for summary of key findings.

**Table 2:** Key findings on the mediating effect of maternal mental health on child mental health

Defined parameters	*b*	SE	90% CI		β	*p*
Direct effect	-0.030	0.005	[-0.039,	-0.020]	-0.084	0.0001
Indirect effect (8mos)	0.006	0.002	[0.003,	0.009]	0.016	0.0001
Total effect	-0.022	0.005	[-0.032,	-0.012]	-0.062	0.0001

*CI* Confidence interval

#### Mediating Effect of Maternal Mental Health on Child Cognitive Ability

Further to be seen in Fig. 3 is a significant, positive, and relatively large direct effect of SES on child cognitive ability (*b* = 4.171, *SE* = 0.120, 90% CI = [3.936, 4.407], β = 0.485, *p* < 0.0001). Children with higher SES tended to have better scores on the latent factor of cognitive ability. This association was mediated by maternal mental health at the 8 month timepoint (*b* = -0.092, *SE* = 0.042, 90% CI = [-0.175, -0.009], β = -0.011, *p* < 0.05). Whereas parameter estimates for the 1 year 9 months timepoint (*b* = 0.011, *SE* = 0.009, 90% CI = [-0.006, 0.028], β = 0.001, *p* > 0.05) and 2 years 9 months timepoint (*b* = 0.002, *SE* = 0.008, 90% CI = [-0.013, 0.017], β = 0.000, *p* > 0.05) were non-significant. The effect of maternal mental health on child cognitive ability resulted in an attenuated total effect of SES on child cognitive ability (*b* = 4.092, *SE* = 0.122, 90% CI = [3.853, 4.331], β = 0.476, *p* < 0.0001). Please see Table 3 for summary of key findings.

**Table 3:** Key findings on the mediating effect of maternal mental health on child cognitive ability

Defined parameters	*b*	SE	90% CI		β	*p*
Direct effect	4.171	0.120	[3.936,	4.407]	0.485	0.0001
Indirect effect (8mos)	-0.092	0.042	[-0.175,	-0.009]	-0.011	0.05
Total effect	4.092	0.122	[3.853,	4.331]	0.476	0.0001

*CI* Confidence interval

## Discussion

The present study sought to determine whether maternal mental health acts as a longitudinal mediator for the association between SES and childhood developmental outcomes. Secondly, we aimed to determine whether there are sensitive periods wherein this mediating effect, if present at all, is particularly pronounced. Our design included both measures of mental health and cognition, allowing for the possibility that the effect and timing of maternal mental health may be somewhat domain specific. There are a number of key findings. First, in this relatively affluent sample, SES has a differential association with child mental health and cognitive ability. Second, maternal mental health mediates both of these relationships – the direct relationship between SES and outcome drops significantly when maternal mental health is considered. Third, in both cases this mediation happens early, largely before the child’s first birthday. Fourth, the mediation varies in size depending upon the outcome domain.

### SES, Maternal Mental Health, and Child Mental Health

In our data, SES was positively associated with maternal mental health; higher SES levels were associated with better maternal mental health. However, the opposite was found for child mental health, with higher SES levels resulting in poorer child mental health. This association was mediated by maternal mental health. The longitudinally mediating effect was primarily accounted for at 8 months. Whilst this mediating effect was relatively small, it suggests the importance of early maternal mental health for critical periods of developmental timing.

Why is higher SES associated with poorer child mental health? It is important to note that our sample was relatively affluent, with a low percentage of participants from low-income households (12.5%). Thus, the majority of the sample were positioned at the higher end of the socioeconomic spectrum. This may be important in interpreting the counterintuitive effect of a negative association between SES and child mental health. Children from affluent backgrounds have been shown to manifest greater anxiety and depression due to parental pressures to achieve at an academic and extracurricular level, as well as isolation due to parental career obligations and intensive schedules (Luthar, 2003; Luthar & Becker, 2002; Parenteau et al., 2020). Considering the sample characteristics and child mental health measures used, it is possible a similar effect is present in our data. This effect is overall mediated by maternal mental health, with better maternal mental health weakening the negative relationship between SES and child mental health. But the effect size of this mediation is relatively small. One possibility is that the mediation effect is relatively small simply because the SES-outcome relationship is relatively small for child mental health. Our SES measure primarily captures the economic circumstances of the family, which may itself be less strongly associated with child mental health, relative to other forms of early life adversity (McLaughlin et al., 2021; Sheridan & McLaughlin, 2014; Sheridan et al., 2020).

### SES, Maternal Mental Health, and Child Cognitive Ability

In contrast to child mental health outcomes, SES had a relatively large *positive effect* on child cognitive ability (Gignac & Szodorai, 2016); higher SES was associated with better child cognitive ability. Again, it is important to highlight the differential relationships between SES and our developmental outcomes. In our data, socioeconomic disadvantage had a stronger and positive relationship with child cognitive performance, relative to the child’s mental health, as might be predicted by the dimensional account of early life adversity (McLaughlin et al., 2021; Sheridan & McLaughlin, 2014). According to this account, deprivation is thought to have a broad and pervasive impact on child development, via the absence of expected, cognitive, linguistic, and social input. The consequence of this is thought to be primarily observed in terms of widespread neural network formation (Johnson et al., 2021) and cold cognitive performance measures (Sheridan et al., 2020).

The SES-cognition association was also mediated by maternal mental health. Taking maternal mental health into consideration reduces the strength of the direct association between SES and child cognitive ability. As was the case with child mental health, this mediating effect was most influential when children were 8 months old, further demonstrating the important role of a child’s proximal environment during the first year of life. The mediating role of maternal mental health could be explained in part by parental buffering and its cascading effects across development. For example, positive parenting behaviour buffers children’s emotional and stress reactivity profiles (Brown et al., 2020; Oppenheimer et al., 2016), which in turn influences cognitive ability (Bell & Wolfe, 2004; Bell et al., 2019; Walle et al., 2022; Wass, 2018). Thus, the early benefits of parental buffering may have led to the reduced SES-cognition association. Put simply, whilst early socioeconomic deprivation might reflect reduced cognitive, social, or linguistic stimulation, this can, to some extent, be buffered by maternal mental health.

### Limitations and Future Directions

Irrespective of the strengths in the current study, such as the inclusion of multiple developmental outcomes, its consideration of maternal mental health as a potential mediator, and its longitudinal consideration of developmental periods, it was not without limitations. For instance, measures of child mental health relied on parent reports. Whilst this is common when measuring mental health in this age group, parent reports may have been reflective of parental mental health, as opposed to that of the child. Furthermore, it is possible that the mediating effect of maternal mental health is stronger for certain aspects of SES. For example, even after multiple aspects of SES and genetic confounding are taken into account, household income is specifically linked to risk of developing mental health problems in childhood (Kinge et al., 2021). Therefore, the mediating effect of maternal mental health may also be specific to certain indicators of SES. This may mean that we are slightly over or underestimating the SES associations with our aggregated measure.

The present study had limitations by way of sampling. As mentioned, SES distribution weighted towards the more affluent end of the spectrum, with a low percentage of participants from low-income households (12.5%). Likewise, the ALSPAC cohort display high education levels, with 42.6% of mothers educated to an A level or above (Fraser et al., 2013). Whilst this sample allowed us to identify the mediating role of maternal mental health in the association between SES and key developmental outcomes, as well as highlight the negative correlation between high SES and child mental health, future research should aim to incorporate a more evenly distributed sample in terms of SES. Such research could further elucidate the complexity of these associations.

Future research should further consider additional SES timelines during development. For example, as previously noted, SES is more strongly related with childhood injury during early development, and smoking behaviour during adolescence (Chen et al., 2002). By extension, it is possible that the mediating effect of maternal mental health varies depending upon SES during different periods of development, such as infancy, early childhood, and adolescence. Considering multiple SES timelines during development could shed light on the mediating role of maternal mental health in these associations. Building on this, future research should consider additional developmental outcomes where the mediating role of maternal mental health in SES-outcome associations have not yet been considered, such as language and social-emotional domains (Guhn et al., 2020; Luo et al., 2022).

### Clinical and Health Policy Implications

The findings of the current study have both clinical and health policy implications. For example, our findings call attention to the importance of maternal mental health during early childhood, chiefly within the first year of life. At present, and in line with British Government requirements, a clinical postnatal check is carried out with mother’s six to eight weeks following the birth of their child (British National Health Service Postnatal Review, 2022). This check includes a general discussion about mental health and well-being. We argue for an extension of the current guidelines, to include regular postnatal mental health checks during the first 12-months after a baby’s birth. In view of the role of maternal mental health on the SES-outcome associations, ensuring the mental well-being of mother’s during this critical period is paramount.

## Conclusion

In conclusion, the current study demonstrates that SES has significant longitudinal associations with two domains of child development – mental health and cognitive ability. However, the association is different depending upon the domain. In both cases, maternal mental health mediates the association. The variability of the mediating role of maternal mental health indicates its role is transient and dependent upon the developmental outcome in question. We have emphasised the complexity of SES-outcome associations and add to the current body of literature calling for the consideration of factors in a child’s proximal environment that likely mediate this association, in hopes of identifying potential targets for intervention and prevention (Letourneau et al., 2013; Liu et al., 2020; Reiss et al., 2019).

## Data Availability

Data used in the present study can be accessed via ALSPAC. Please note that the study website contains details of all the data that is available through a fully searchable data dictionary and variable search tool (http://www.bristol.ac.uk/alspac/researchers/our-data/).

## Declarations

### Open Access

To encourage and promote open science, a Preprint of this manuscript has been made available on PsyArXiv (https://psyarxiv.com/4swy8).
